# Surviving historical Patagonian landscapes and climate: molecular insights from *Galaxias maculatus*

**DOI:** 10.1186/1471-2148-10-67

**Published:** 2010-03-08

**Authors:** Tyler S Zemlak, Evelyn M Habit, Sandra J Walde, Cecilia Carrea, Daniel E Ruzzante

**Affiliations:** 1 Department of Biology, Dalhousie University, Halifax Nova Scotia, Canada B3H 4J1; 2 Environmental Science Centre EULA-Chile, Universidad de Concepción, and Centro de Investigaciones en Ecosistemas Patagónicos (CIEP), Chile; 3 Universidad Nacional de Comahue, Quintral 1250, San Carlos de Bariloche, 8400 Rio Negro, Argentina

## Abstract

**Background:**

The dynamic geological and climatic histories of temperate South America have played important roles in shaping the contemporary distributions and genetic diversity of endemic freshwater species. We use mitochondria and nuclear sequence variation to investigate the consequences of mountain barriers and Quaternary glacial cycles for patterns of genetic diversity in the diadromous fish *Galaxias maculatus *in Patagonia (~300 individuals from 36 locations).

**Results:**

Contemporary populations of *G. maculatus*, east and west of the Andes in Patagonia, represent a single monophyletic lineage comprising several well supported groups. Mantel tests using control region data revealed a strong positive relationship when geographic distance was modeled according to a scenario of marine dispersal. (*r *= 0.69, *P = 0.055*). By contrast, direct distance between regions was poorly correlated with genetic distance (*r *= -0.05, *P *= 0.463). Hierarchical AMOVAs using mtDNA revealed that pooling samples according to historical (pre-LGM) oceanic drainage (Pacific vs. Atlantic) explained approximately four times more variance than pooling them into present-day drainage (15.6% vs. 3.7%). Further *post-hoc *AMOVA tests revealed additional genetic structure between populations east and west of the Chilean Coastal Cordillera (coastal vs. interior). Overall female effective population size appears to have remained relatively constant until roughly 0.5 Ma when population size rapidly increased several orders of magnitude [100× (60×-190×)] to reach contemporary levels. Maximum likelihood analysis of nuclear alleles revealed a poorly supported gene tree which was paraphyletic with respect to mitochondrial-defined haplogroups.

**Conclusions:**

First diversifying in the central/north-west region of Patagonia, *G. maculatus *extended its range into Argentina via the southern coastal regions that join the Pacific and Atlantic oceans. More recent gene flow between northern populations involved the most ancient and most derived lineages, and was likely facilitated by drainage reversal(s) during one or more cooling events of the late Pleistocene. Overall female effective population size represents the end result of a widespread and several hundred-fold increase over approximately 0.5 Ma, spanning several climatic fluctuations of the Pleistocene. The minor influence of glacial cycles on the genetic structure and diversity of *G. maculatus *likely reflects the access to marine refugia during repeated bouts of global cooling. Evidence of genetic structure that was detected on a finer scale between lakes/rivers is most likely the result of both biological attributes (i.e., resident non-migratory behavior and/or landlocking and natal homing in diadromous populations), and the Coastal Cordillera as a dispersal barrier.

## Background

The past two decades of phylogeographic research have tremendously increased our understanding of the evolutionary influences of Quaternary geological and climatic events on endemic biodiversity, but research thus far has been severely biased to regions of the Northern Hemisphere [1]. Among the most neglected regions of the Southern Hemisphere is South America. This finding is surprising considering that the tropics of South America are expected to host an unmatched level of species richness and represent one of the best natural laboratories for studying speciation. Only recently has the Amazonian rainforest been the focus of large-scale surveys of intra-specific genetic variation aimed at understanding the potential mechanisms contributing to, and maintaining such high levels of species diversity [2,3].

Phylogeographic patterns in temperate South America have also received relatively little attention, but recent studies point to the over-riding importance of two historic variables: the Andean mountain orogeny (starting approximately 23 Ma [4]) and the glacial cycles of the Quaternary (2.5 Ma - 10,000 bp). Estimated rates of trans-Andean gene flow vary greatly among taxa, including one species of plant [5], two fishes [6,7], and several mammals [8-10], but the continental divide represents a barrier for all species examined to date. Post-glacial patterns of dispersal for several taxa indicate the existence of several independent Quaternary glacial refugia east of the Andes on the Patagonian Steppe [6,7,9,11-13], and west of the Andes, both within [7,14] and outside of northern and western limits of the glaciers [6,7,12,14-16]. Populations persisting in some of these refugia experienced repeated founder-flush cycles leading to the purging of genetic variance and resulting in the development of genetic structure [17].

Phylogeographic patterns of aquatic organisms are largely determined by historical changes in hydrological landscapes. In Patagonia, starting approximately 23 Ma [4], the rise of the southern Andes created a continental divide that separated eastern and western basins into primarily Atlantic and Pacific drainages, and studies of two freshwater species, *Percichthys trucha *[6] and *Galaxias platei *[7] have shown that the uplift presented a significant barrier to gene flow. Glacial advances during periods of global cooling in the Quaternary eliminated much southern and higher altitude aquatic habitat [18,19], reducing and/or displacing populations. Patterns of intra-specific genetic diversity of fishes and crabs suggest that multiple refugia existed to the north and east of continental ice, as well as within glacial margins of the central-west [6,7,14]. Important rearrangement of basins also occurred during the retreat of continental glaciers in Patagonia [20]. The formation of temporary but large proglacial lakes at the fringes of melting glaciers, coupled with high altitude mountain run-off, probably allowed the exchange of aquatic organisms among drainage basins, and allowed the expansion of populations into unoccupied basins [7,14]. Catastrophic shifts in drainage direction (from Atlantic to Pacific) in several basins, as ice dams collapsed during the retreat of glaciers, have been implicated as important vectors of gene flow from E → W across the Andean divide for *G. platei *[7,20].

In the present study, we examined the potential consequences that major Quaternary geological and climatic events in Patagonia had on the evolution of *Galaxias maculatus*, one of the most widespread diadromous fish species worldwide. We employed DNA sequence variation at both mitochondrial and nuclear loci from *G. maculatus *populations throughout their range in Patagonia to test for influences of mountain ranges as barriers to gene flow and the roles of repeated Pleistocene glacial cycles in further shaping the contemporary genetic diversity and structure of a diadromous fish. To the best of our knowledge, this study represents the first phylogeographic investigation of a diadromous fish species in temperate South America.

*Galaxias maculatus *is considered amphidromous, a special case of catadromy characterized by a shortened duration at sea. Disjunct populations of *G. maculatus *are found in the coastal systems of southern Australia, Tasmania, New Zealand and surrounding islands (e.g. Lord Howe, Chattam, New Caledonia), and in the temperate latitudes of South America and the Malvinas (Falkland) Islands [21-24]. Although both dispersal [25,26] and vicariance [22,27] hypotheses have been proposed to explain this fragmented distribution, most empirical evidence indicates that populations dispersed from Australia to other locations by ocean currents (West Wind Drift; herein WWD) [28-31].

**Figure 1:**
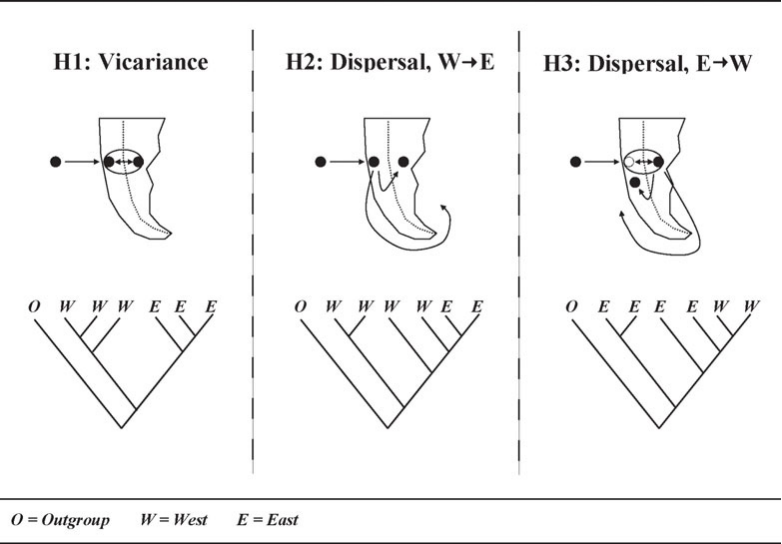
**Alternative colonization histories for *Galaxias maculatus *in Patagonia, South America**.

The influence of the Andes mountains in shaping the genetic structure of *G. maculatus *in Patagonia is uncertain. One possibility is that a widespread distribution was established prior to the onset of Andean orogeny and vicariance either temporarily, or permanently isolated eastern and western populations to create deep genetic structure (vicariance). Equally likely is the establishment of a widespread distribution following Andean uprise. Ancestral lineages could have persisted in either eastern or western Patagonia and subsequently expanded into the adjacent region (dispersal, W → E or E → W). To better understand the colonization history of the surviving lineage(s) that comprise contemporary South American populations of *G. maculatus *we reconstructed the phylogenetic relationships of approximately 300 individuals with respect to a suitable outgroup. Vicariance assumes a widespread distribution prior to Andean uprise, followed by a simultaneous division of eastern and western locations to yield reciprocally monophyletic haplotype assemblages. Alternatively, dispersal suggests that *G. maculatus *attained its widespread distribution throughout Patagonia following Andean orogeny to imply that one location, either east or west (depending on dispersal direction), was colonized prior to the establishment of populations on both sides of the Andes. Phylogenetically, this would be represented by the dispersing lineage being nested within the source lineage. Assuming that *G. maculatus *originally arrived in South America via WWD, a scenario involving E → W dispersal would suggest local extirpation of western populations followed by recolonization of an eastern lineage (Figure 1).

In the event that dispersal was an important component of colonization history, there are at least two mechanisms that could have facilitated connections between eastern and western Patagonia. First, the diadromous capacity of *G. maculatus *affords an indirect route of marine-mediated stepping-stone dispersal. Populations could have invaded from either eastern or western sources by accessing the connection between Atlantic and Pacific oceans at the southernmost tip of South America. The second mechanism involves drainage reversals; a climate-induced shift in which formerly Atlantic draining river systems catastrophically shifted to assume an opposite direction of outflow into the Pacific. Drainage reversals following the Last Glacial Maximum (LGM) are thought to have facilitated a direct route of dispersal across the Andean divide for the co-distributed freshwater species *G. platei *and could have had similar implications for *G. maculatus*. Assuming that colonization history reflects dispersal, we attempt to discern between the relative influences of direct (trans-Andean) versus indirect (marine) modes of dispersal by comparing genetic distance with geographic distance modeled under each scenario. Furthermore, we test for the specific influences of drainage reversals following the LGM by comparing partitions of genetic variation according to the expected pre- and post-LGM drainage patterns in Patagonia.

Finally, we examined the potential influence of repeated Pleistocene glacial cycles on long-term effective population size. The diadromous capacity of *G. maculatus *affords populations an additional and/or alternative form of refuge in the much more stable marine realm. The availability of marine refugia might have reduced, or exempted populations from the founder-flush cycles that are typically experienced by freshwater-limited taxa [32-34], and the reshuffling effects of drainage reversals. However, exceptions to an amphidromous life-cycle have been reported for several populations in South America in the form of physically landlocked and non-migratory populations that remain exclusively within freshwater throughout their life cycle. Depending on the duration and frequency with which some populations remained within freshwater in the past, glacial cycles could have introduced new genetic structure and reduced population sizes throughout parts of the range of *G. maculatus*. Bayesian skyline plots are employed to reconstruct long-term historical effective population sizes over a period of repeated Pleistocene glacial advances.

## Methods

### Sample collection and preservation

**Figure 2:**
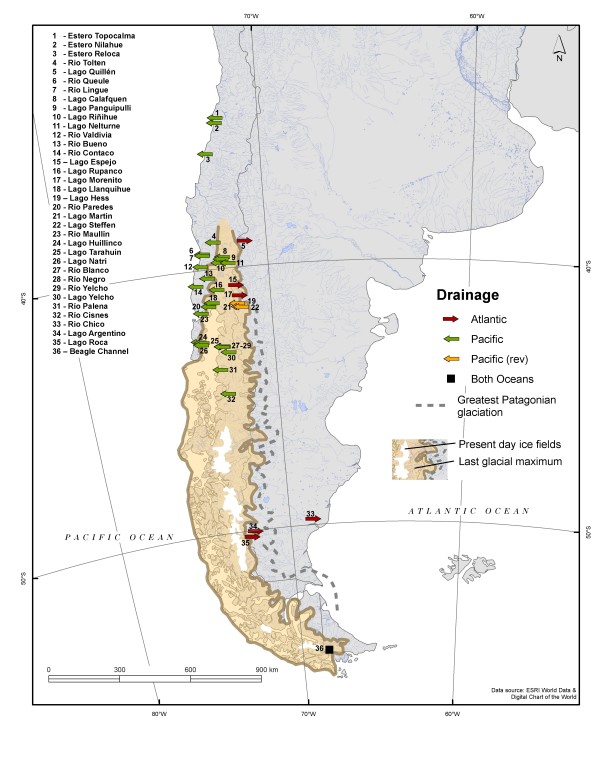
**Collection locations for *Galaxias maculatus *throughout Patagonia, South America**. Sampled locations are represented by arrows that indicate contemporary drainge direction, either Atlantic or Pacific. Pacific (rev) denotes a drainage reversal, from Atlantic to Pacific, which likely occurred following the retreat of Pleistocene glaciers [20]. The extent of the LGM and contour of the largest Patagonian glaciation were adapted from [18,20].

A total of 299 individuals were collected using seine nets and electrofishing between 1998-2007 from 36 lakes and rivers throughout Argentina and Chile (Figure 2). Sample sizes varied according to location and region (Table 1). Ninety (30%) individuals derived from 10 Argentinean lakes, and the remaining 209 individuals were collected from 26 Chilean lakes/rivers. Sub-samples (gill, muscle, fin, blood) for molecular analyses were fixed in 95% ethanol.

**Table 1:** Collection site details for Patagonian lakes/rivers, categorized according to relative position with the Andes, either west (Chile) or east (Argentina), and therein ordered in a north/south orientation with respect to latitude.

					AMOVA Classifications						
								
Collection Site	#Indv.	Basin	Latitude (dd mm ss)	Longitude (dd mm ss)	Basin Origin	**Collection Loc**.	Ocean Drainage		Mantel Groups	Sequence Diversity	
									
							Current	Ancient		*h*	*π*
***Chile***											
Estero Topocalma	1	Topocalma	34 17 51	71 57 46	Coastal	Coastal	Pacific	Pacific	NW	-	-
Estero Nilahue	4	Nilahue	34 29 01	72 00 58	Coastal	Coastal	Pacific	Pacific	NW	1.0000 +/- 0.1768	0.014429 +/- 0.009937
Estero Reloca	3	Reloca	35 37 49	72 33 45	Coastal	Coastal	Pacific	Pacific	NW	1.0000 +/- 0.2722	0.015038 +/- 0.011755
Rio Tolten	8	Tolten	38 59 09	72 37 10	Andean	Andean	Pacific	Pacific	W1	0.8929 +/- 0.1113	0.028831 +/- 0.016213
Rio Queule	10	Queule	39 23 30	73 11 57	Coastal	Coastal	Pacific	Pacific	W1	1.0000 +/- 0.0447	0.016423 +/- 0.009146
Rio Lingue	10	Lingue	39 26 45	73 12 48	Coastal	Coastal	Pacific	Pacific	W1	1.0000 +/- 0.0447	0.030121 +/- 0.016386
Lago Calafquen	10	Valdivia	39 34 16	72 14 27	Andean	Andean	Pacific	Pacific	W1	1.0000 +/- 0.0447	0.012898 +/- 0.007281
Lago Pangupulli	5	Valdivia	39 38 41	72 19 24	Andean	Andean	Pacific	Pacific	W1	1.0000 +/- 0.1265	0.009045 +/- 0.005973
Lago Rinihue	10	Valdivia	39 46 29	72 27 10	Andean	Andean	Pacific	Pacific	W1	0.9333 +/- 0.0773	0.033125 +/- 0.017973
Lago Neltume	10	Valdivia	39 48 36	71 59 40	Andean	Andean	Pacific	Pacific	W1	0.9778 +/- 0.0540	0.009464 +/- 0.005462
Rio Valdivia	9	Valdivia	39 51 46	73 21 12	Andean	Coastal	Pacific	Pacific	W1	1.0000 +/- 0.0524	0.004038 +/- 0.002605
Rio Bueno	10	Bueno	40 19 27	73 05 38	Andean	Andean	Pacific	Pacific	W1	0.9333 +/- 0.0620	0.026235 +/- 0.014330
Rio Contaco	4	Contaco	40 34 44	73 41 53	Coastal	Coastal	Pacific	Pacific	W1	1.0000 +/- 0.1768	0.035297 +/- 0.023563
Lago Rupanco	8	Bueno	40 47 23	72 41 11	Andean	Andean	Pacific	Pacific	W1	1.0000 +/- 0.0625	0.005026 +/- 0.003194
Lago Llanquihue	10	Maullin	41 15 43	72 59 40	Andean	Andean	Pacific	Pacific	W1	1.0000 +/- 0.0447	0.029946 +/- 0.016294
Rio Paredes	5	Maullin	41 23 14	73 11 16	Andean	Coastal	Pacific	Pacific	W1	1.0000 +/- 0.1265	0.027945 +/- 0.017431
Rio Maullin	10	Maullin	41 36 43	73 36 22	Andean	Coastal	Pacific	Pacific	W1	0.8667 +/- 0.01072	0.006099 +/- 0.003673
Lago Huillinco	7	Chiloe	42 40 16	73 54 11	-	-	Pacific	Pacific	W2	1.0000 +/- 0.0764	0.031567 +/- 0.018129
Lago Tarahuin	10	Chiloe	42 43 01	73 45 01	-	-	Pacific	Pacific	W2	1.0000 +/- 0.0447	0.013705 +/- 0.007706
Lago Natri	10	Chiloe	42 47 60	73 47 18	-	-	Pacific	Pacific	W2	1.0000 +/- 0.0447	0.008104 +/- 0.004739
Rio Blanco	9	Blanco	42 56 04	72 43 28	-	-	Pacific	Pacific	W2	1.0000 +/- 0.0524	0.018287 +/- 0.010272
Rio Negro	10	Yelcho	42 56 35	72 40 60	-	-	Pacific	Pacific	W2	1.0000 +/- 0.0447	0.014248 +/- 0.007995
Rio Yelcho	7	Yelcho	42 57 18	72 45 08	-	-	Pacific	Pacific	W2	1.0000 +/- 0.0764	0.030949 +/- 0.017784
Lago Yelcho	9	Yelcho	43 10 60	72 25 53	-	-	Pacific	Pacific	W2	1.0000 +/- 0.0524	0.023038 +/- 0.012821
Rio Palena	10	Palena	43 48 27	72 57 45	-	-	Pacific	Pacific	W2	1.0000 +/- 0.0447	0.015837 +/- 0.008836
Rio Cisnes	10	Cisnes	44 44 48	72 42 20	-	-	Pacific	Pacific	W2	1.0000 +/- 0.0447	0.014202 +/- 0.007970
											
***Argentina***											
Lago Quillen	10	Limay	39 02 00	71 02 00	-	-	Atlantic	Atlantic	E	0.9778 +/- 0.0540	0.049779 +/- 0.026769
Lago Espejo	9	Limay	40 41 00	71 40 00	-	-	Atlantic	Atlantic	E	1.0000 +/- 0.0524	0.015038 +/- 0.008530
Lago Morenito	2	Limay	41 05 00	71 32 00	-	-	Atlantic	Atlantic	E	1.0000 +/- 0.5000	0.010025 +/- 0.010633
Lago Hess	9	Manso	41 22 02	71 44 23	-	-	Pacific	Atlantic	E	1.0000 +/- 0.0524	0.013506 +/- 0.007708
Lago Martin	9	Manso	41 30 00	71 40 00	-	-	Pacific	Atlantic	E	0.889 +/- 0.0910	0.017648 +/- 0.009931
Lago Steffen	13	Manso	41 31 00	71 33 00	-	-	Pacific	Atlantic	E	0.9872 +/- 0.0354	0.010732 +/- 0.005965
Rio Chico	9	Rio Chico	49 47 00	68 38 00	-	-	Pacific	Atlantic	SE	1.0000 +/- 0.0524	0.029307 +/- 0.016181
Lago Argentino	10	Santa Cruz	50 02 05	72 04 00	-	-	Atlantic	Atlantic	SE	0.8667 +/- 0.1072	0.010091 +/- 0.005795
Lago Roca	9	Santa Cruz	50 31 33	72 41 56	-	-	Atlantic	Atlantic	SE	1.0000 +/- 0.0524	0.013033 +/- 0.007456
Beagle Channel	10	-	54 47 43	68 15 28	-	-	Atlantic	Atlantic	SE	1.0000 +/- 0.0447	0.018296 +/- 0.010136
								
**36**	**299**	**19**								**0.9989 +/- 0.0005**	**0.046394 +/- 0.022376**

The northernmost Chilean locations are also classified according to relative position with the Coastal Cordillera, either west (Coastal) or east (Interior). Details include the number of individuals sampled, local drainage basin membership (basin), GPS coordinates (latitude/longitude), and expected direction of ocean drainage before (ancient) and after (current) the last glacial maximum [7,18,20], and location of headwaters as either coastal or Andean for a sub-set of Chilean locations used for *post-hoc *AMOVA analysis (Table 2). Sequence diversity metrics [haplotype (*h*) and nucleotide (*π*)] based on mitochondrial control region sequence data are listed for each location.

### Sequence Data

Prior to DNA extraction all tissue samples were dried of ethanol by exposure to ambient temperature for approximately 120 min. Total genomic DNA was isolated from the majority of sub-samples (10 μL of blood or 2 × 2 mm^2 ^tissue) using the glassmilk procedure described in [35] with slight modifications for execution using a MultiPROBE^® ^II HT PLUS EX robotic liquid handling system (PerkinElmer). Sub-samples derived from 2 Argentinean locations (Quillén, Espejo) showed signs of tissue degradation and were extracted using standard phenol/chloroform protocols, and subsequently concentrated using ethanol precipitation [36].

A section of approximately 800 bp of the mitochondrial control region was amplified from all Chilean samples and the majority of Argentinean individuals using the primer combination *S-phe *5'-GCT TTA GTT AAG CTA CG-3' [37] and *P3 *5'-AAC TTC CAT CCT CAA CTC CCA AAG-3' [38]. The full mitochondrial genome available in GenBank (NC_004594) was used to design species specific forms of both *S-phe *(5'-GCT TTA CTT AAG CTA CG-3') and *P3 *(5'-AAC TCT CAC TCT TAA CTC CCA AAG-3') to amplify all individuals from 6 Argentinean locations (lakes Quillén, Espejo, Morenito, Hess, Martin, Steffen). PCR reactions were 25 μL: 2.5 μL 10× Reaction Buffer [100 mM KCl, 100 mM (NH_4_)_2_SO_4_, 200 mM Tris HCl (pH 8.75, 22°C), 1% Triton X-100, 1 mg/ml BSA], 2.5 μL dNTPs (2 μM each), 3.75 μL MgSO_4 _(20 mM), 13 μL ddH_2_O, 0.25 μL of each 10 μM primer, 1 U of Tsg Polymerase (Bio Basic Inc.) and 2.0-2.5 μL of DNA template. A Mastercycler^® ^EP Gradient (Eppendorf) thermal cycler was used to conduct all reactions under the following thermal regime: an initial denaturing temperature of 94°C for five min, followed by 35 cycles of 94°C for 1 minute, 50°C for 1 minute 30 seconds, and 72°C for 1 minute 30 seconds, and a final extension at 72°C for 5 min.

Nuclear DNA (approximately 600 bp) was also amplified from a subset of individuals (N = 26) selected based on *post-hoc *information to represent each mitochondrial haplogroup. These individuals represented rivers Bueno (4), Valdivia (3), and Toltén (2) and lakes Llanquihue (4), Tarahuin (5), and Natri (3) in Chile, and lakes Argentino (4) and Martin (1) in Argentina. The target locus was isolated using previously unpublished primer sequences [ANL22 forward (5'-TGT TTG GCT TCT ATG CAG GA-3') and reverse (5'-TGC GAT CCA TCA TCA ACT TT-3')] which originated from a genomic library aimed at isolating anonymous nuclear loci from a closely allied species *G. platei *following protocols of [39,40]. Except for an annealing temperature of 56°C, the PCR conditions used to amplify the nuclear locus were identical to those reported above for control region data.

Amplicons were visualized using 1.0% agarose gels and sent off site for bidirectional DNA sequencing at Macrogen Inc. locations in Korea and the USA. Each forward and reverse sequence was edited using Sequencher™. Sequences were aligned in ClustalX2 [41] using default parameters and subsequently checked by eye. The complete alignment for control region data was compressed into haplotypes using DnaSP 3.0 [42] and listed under the following accessions in GenBank: GQ180504-GQ180776. Ambiguous nuclear alleles, represented by sequences containing 2 or more heterozygous base calls, were separated with 100% probability using the program PHASE v2.1.1 [43]. All alleles were confirmed to be free of recombination using both RDP and MaxChi algorithms as executed in the program RDP3 [44], and subsequently blasted in GenBank to ensure no matches with mitochondrial DNA. Nuclear allele sequences are available in GenBank under the following accessions: GQ180467-GQ180503.

### Data Analysis (control region mtDNA)

Standard molecular diversity indices for control region haplotypes [haplotype diversity (*h*), nucleotide diversity (*π*), nucleotide frequencies, transition/transversion ratio, number of polymorphic sites] were calculated using Arlequin 3.1 [45]. Phylogenetic relationships among haplotypes were reconstructed using maximum likelihood methods as implemented in PHYML [46] using the web based server PHYML-Online [47]. Modeltest [48] selected the GTR+G (γ = 0.5483) as the most appropriate model of molecular evolution for phylogenetic analysis with branch support based on 100 bootstrap replicates. The outgroup was based on a single Tasmanian *G. maculatus *control region haplotype (NC_004594) because it represented the only full length control region isolate available in GenBank that fully overlapped with Patagonian derived haplotypes. However, auxiliary phylogenetic analyses were conducted using partial isolates of control region sequences from [30] (AF240184-AF240339) to justify the use of a single sequence as an outgroup. Maximum likelihood analysis of ~150 haplotypes derived from Tasmanian and New Zealand origins indicate close associations between all haplotypes derived from eastern Pacific sources and support Patagnonian haplotypes as a strongly supported monophyletic group. Supplemental trees are available in newick tree format as Additional files 1, 2.

Alternate dispersal scenarios were investigated using Mantel Tests as implemented in Arelquin 3.1 [45] using 1000 bootstrap replicates. Collection locations were grouped into regional categories based on both proximity and location with respect to the Andes (Table 1): north west (NW), west 1 (W1), west 2 (W2), east (E) and south east (SE). Approximate pairwise distances (km) between regions were calculated from the central geographic point using Google Earth according to two models: first, a direct route across terrestrial landscape to represent trans-Andean dispersal; second, an indirect route to circumscribe the southern Andes via oceanic connections to represent marine-mediated dispersal (Additional file 3). Genetic distances between regions were based on standard pairwise estimates of *F*_*ST *_calculated using Arlequin 3.1.

**Figure 3:**
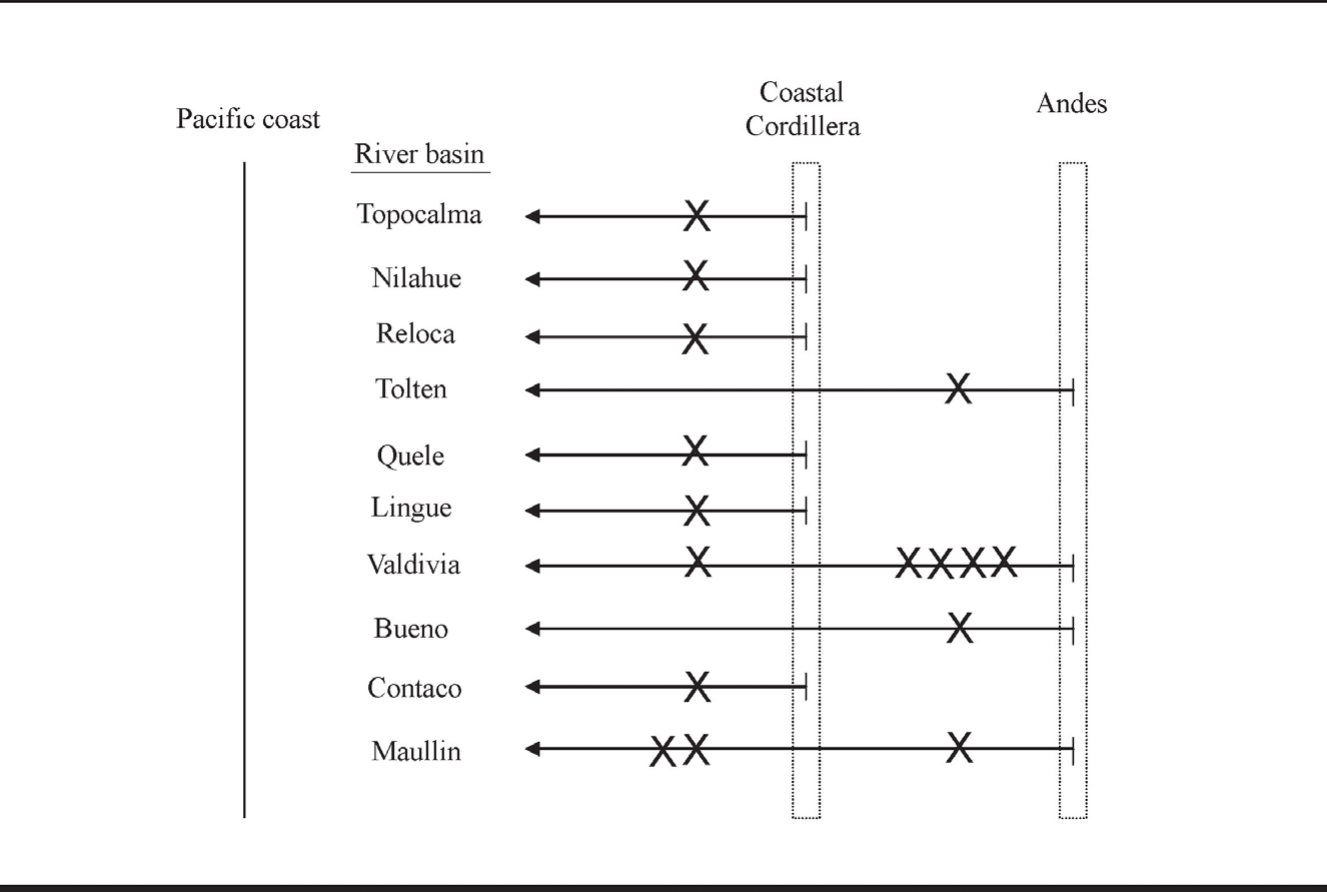
**Sampling regime for *post-hoc *AMOVA analysis conducted for a subset of collection locations in Chile between latitudes 34°S and 40°S**. We grouped each population as "coastal" or "Andean" for AMOVA analysis based on two criteria: (2A) location of headwaters and (2B) location of collection site (Table 1). The diagram depicts the number of collection locations (X) that are coastal versus Andean.

Hierarchical Analysis of Molecular Variance (AMOVA), as executed in Arelquin 3.1 [45], was used to determine how contemporary genetic variation is partitioned over the range of *G. maculatus*. Groupings included (1A) lake/river of collection, (1B) river drainage, (1C) present-day and, (1D) ancient (pre LGM) ocean (Pacific or Atlantic) drainage. Further *post-hoc *regional analysis was conducted for a subset of collection locations in Chile between latitudes 34°S and 40°S. This regional consideration was motivated by distributional trends of haplotype groups which suggested compositional differences between populations located near the Pacific coast versus those located within the continental interior of the Andean foothills (see *Costal Cordillera *in results section). The analysis involved 17 collection sites from 10 river basins, all of which drain into the Pacific Ocean (Table 2, Figure 3). Running in a north to south orientation are two mountain ranges: the Coastal Cordillera located near the Pacific coast, and the Andean mountains to the east with the Central Valley in between the two mountain chains. Of the 10 river basins that were sampled, 4 originate in the Andean foothills and bisect the Central Valley and the Coastal Cordillera en route to draining into the Pacific (Tolten, Valdivia, Bueno, Maullin), two of which were represented by collection sites located on either side of the Coastal Cordillera (Valdivia, Maullin). The remaining 6 basins originate within the Coastal Cordillera and extend only a short distance to the coast (Topocalma, Nilahue, Reloca, Queule, Lingue, Contaco). We grouped each population as "coastal" or "Andean" for AMOVA analysis based on two criteria: (2A) location of headwaters and (2B) location of collection site (Table 1).

**Table 2:** Analysis of molecular variance (AMOVA) using mitochondrial control region sequences for 299 individuals of *Galaxias maculatus *collected over the study area (all samples) and a subset of locations from northern Chilean Patagonia (90 individuals).

	**% of variation (*df*)**		
	
**Grouping**	**Among groups**	**Among populations within groups**	**Within populations**
*All samples*			
**1A. Lake/River**	61.00 *(35)*	-	39.00 *(263)*
**1B. River drainage system**	29.94 *(18)*	32.08 *(16)*	37.98 *(254)*
**1C. Current ocean drainage**	3.68 *(1)*	58.76 *(33)*	37.55 *(254)*
**1D. Ancient ocean (pre-LGM) drainage**	15.55 *(1)*	49.41 *(33)*	35.04 *(254)*
			
*Chile*			
**2A. River basin headwaters**	6.64 *(1)*	58.06 *(15)*	35.3 *(110)*
**2B. Collection location**	13.93 *(1)*	51.73 *(15)*	34.34 *(110)*

All samples were grouped according to (A) lake/river from which individuals were collected, (B) the freshwater basin containing each lake/river, and ocean (Pacific or Atlantic) into which each basin, (C) currently drains, or (D) histocially drained [i.e. ancient refers to the conditions before the last glacial maximum (LGM) approximately 10,000-20,000 BP]. Further *post-hoc *regional analysis was conducted for a subset of collection locations in Chile between latitudes 34°S and 40°S. We grouped each population as "coastal" or "Andean" for AMOVA analysis based on two criteria: (2A) location of headwaters and (2B) location of collection site (Table 1). Variation is reported as a percentage of the total with degrees of freedom (d.f.).

Historical population sizes of *G. maculatus *were estimated using the coalescent-based Bayesian skyline plot [49] as implemented in the program BEAST 1.4 [50]. Priors included the GTR+G+I model of molecular evolution with an estimated substitution rate matrix (A-C = 1.35, A-G = 7.83, A-T = 0.34, C-G = 1.02, C-T = 3.47, G-T = 1.0) as determined by Modeltest [48], and implemented a mutational timescale of 0.01876 substitutions/site/My appropriate for galaxiids [51]. Starting operators were based on default settings and auto-optimized during searches of parameter space using Markov Chain Monte Carlo (MCMC) sampling procedures. Parameter estimates were based on posterior probability distributions constructed by sampling the stationary distribution for 40,000,000 generations, sampling every 1000 steps.

### Data Analysis (nuclear locus)

Standard molecular indices were calculated as per the mtDNA control region. Unrooted phylogenetic relationships among alleles were reconstructed using maximum likelihood methods as implemented in PHYML [46] using the web based server PHYML-Online [47]. Modeltest [48] selected the K81uf+G (γ = 0.5759) as the most appropriate substitution model. Branch support was based on 100 bootstrap replicates.

## Results

A total of 273 mtDNA control region haplotypes were identified from 299 individuals of *G. maculatus *collected from throughout the species distribution in South America (Additional file 4). Overall nucleotide frequencies were: A (28.94%), C (24.30%), G (19.36%), T (27.41%). The total number of polymorphic sites was 250, with a transition/transversion ratio of 2.16, 92 observed indels, an average of 37.71 +/- 16.44 pairwise differences between sequences (4.6 +/- 2.0%), a nucleotide diversity (*π*) of 0.9989 +/- 0.0005 and haplotype diversity (*h*) of 0.046394 +/- 0.022376. Individual nucleotide and haplotype diversity estimates are listed for each location in Table 1, and do not vary according to latitude.

**Figure 4:**
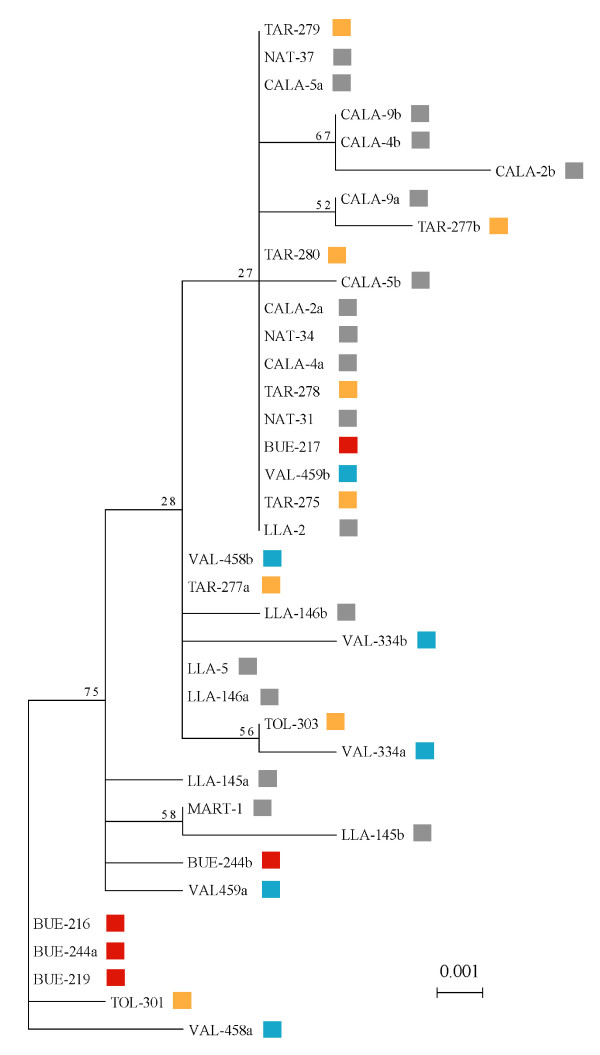
**Unrooted maximum likelihood tree of nuclear haplotypes from 26 individuals**. The subset of individuals screened for the nuclear locus ANL22 were selected to represent the full spectrum of mitochondrial diversity (mitochondrial haplogroups 1-8). Capitalized acronyms in the sequence names indicate the collection location of each individual [Tarahuin (TAR), Natri (NAT), Argentino (CALA), Bueno (Bue), Valdivia (VAL), Llanquihue (LLA), Tolten (TOL), Martin (MART)], numbers represent unique individuals and the letters 'a' and 'b' denote separate alleles of heterozygotes.

Nineteen unique alleles were recovered from the 26 individuals sequenced at the nuclear locus, and over half of the individuals (61%) were homozygous for a given allele. Overall nucleotide frequencies were: 32.7% (A), 17.7% (C), 21.3% (G) and 28.3% (T). The total number of polymorphic sites was 19, with a trasition/transversion ratio of 0.58, no indels, an average of 2.35 +/- 0.002 pairwise differences between sequences, a nucleotide diversity (*π*) of 0.0039 +/- 0.0024 and haplotype diversity (*h*) of 0.8679 +/- 0.0495. Maximum likelihood analysis of alleles revealed a poorly supported gene tree which was paraphyletic with respect to mitochondrial-defined haplogroups (Figure 4).

### Evidence of marine dispersal

**Figure 5:**
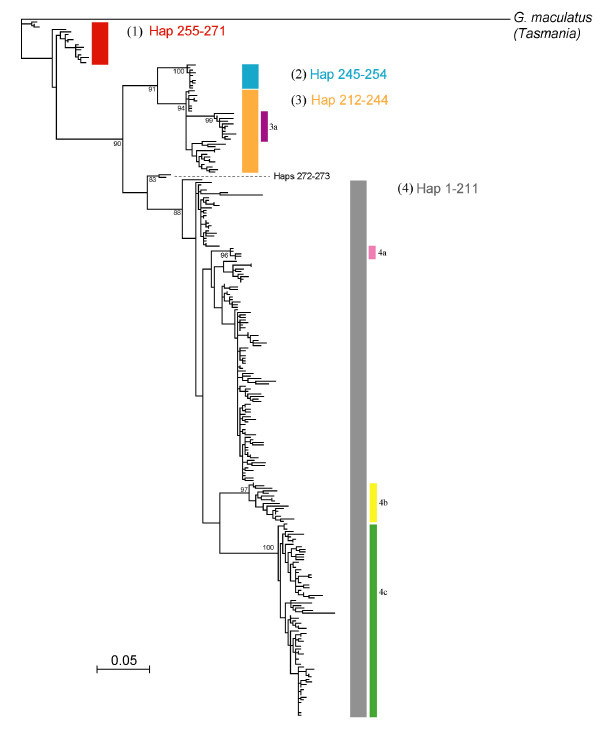
**Maximum likelihood tree of mitochondrial haplotypes rooted with *G. maculatus *(Tasmania)**. Strongly supported haplotype clusters are labeled as haplogroups 1-4. Additional strongly supported branches within haplogroups are identified as sub-groups (a-c). Branch support is based on bootstrap resampling.

Maximum likelihood analysis of mitochondrial control region haplotypes revealed four distinct clusters (Figure 5). The four clusters (herein referred to as haplogroups 1-4) included the following haplotypes: (1) 255-271, (2) 245-254, (3) 212-244, (4) 1-211. Smaller, well supported clusters were also recognized within haplogroups 3 and 4 (herein sub-groups): (3a) 233-243, (4a) 206-210, (4b) 103-118, (4c) 25-100. Haplotypes 272-273 formed a distinct, well-supported branch among basal lineages, but was not recognized as a separate sub-group because of low sampling density (i.e. only consisted of 2 haplotypes).

**Figure 6:**
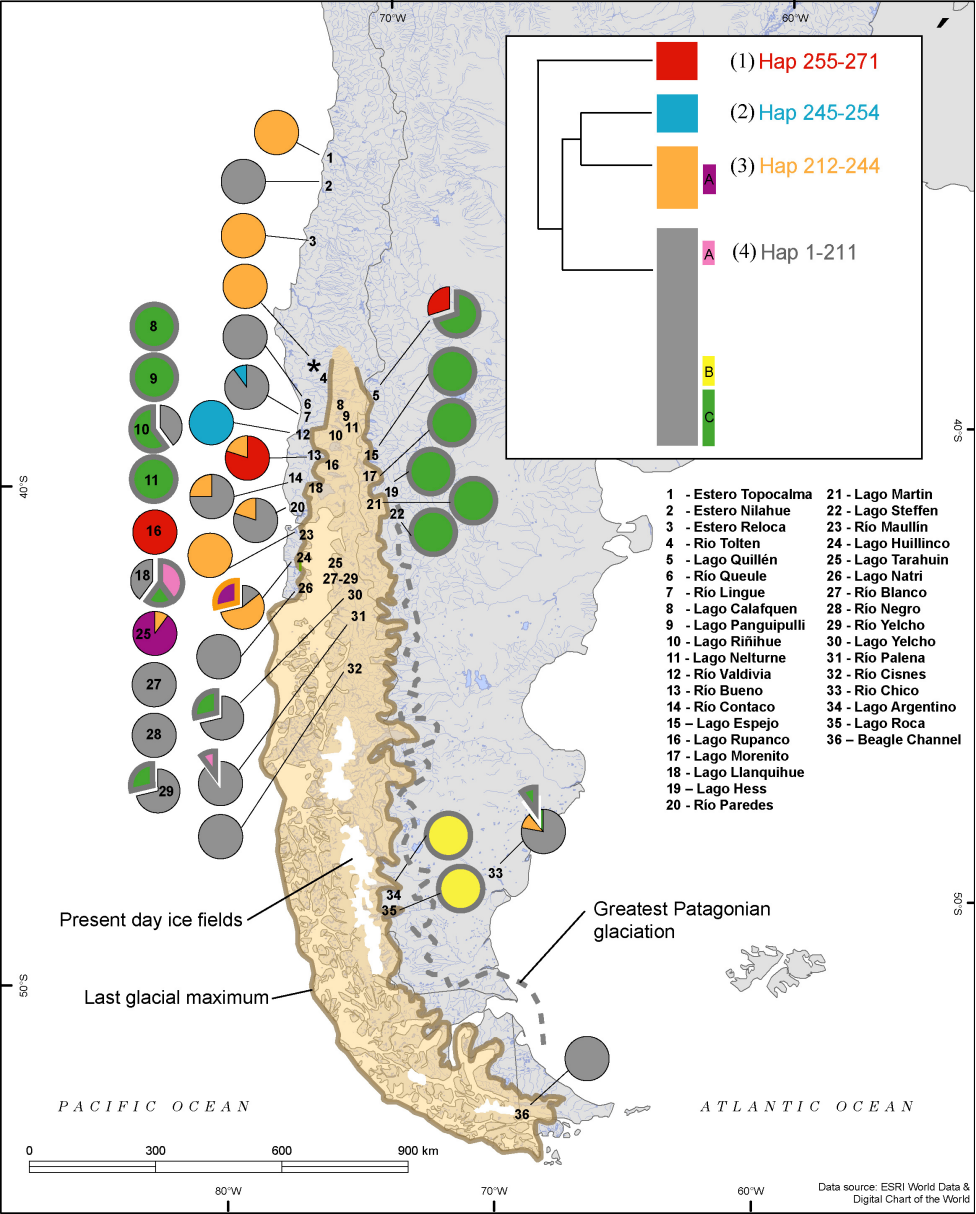
**Frequency distribution of mitochondrial haplotype groups by sampling location**. Haplogroup and sub-group designations follow strongly supported clusters defined in rooted mitochondrial haplotype phylogeny (Fig. 5). Sub-groups are outlined in colours which correspond to the respective haplogroup within which each is nested.

When frequency of each haplogroup at each collection location is plotted by geographic location (Figure 6), we obtain a pattern reflective of dispersal from W → E. Northern Chilean Patagonia hosts the most diverse assemblage of haplotype groups. By contrast, populations in Argentina had mostly haplotypes from sub-groups nested within haplogroup 4; sub-group 4c dominated the northern Andean locations (Limay & Manso river basins) and haplogroup 4b was ubiquitous in southern Andean locations (lakes Roca and Argentino). Haplogroup 1 is primarily limited to the northwest coastal regions of Chile, except for very minor representation in the most northern drainage of Argentina. The most geographically widespread lineage, haplogroup 4, was found in both Chile and Argentina.

**Figure 7:**
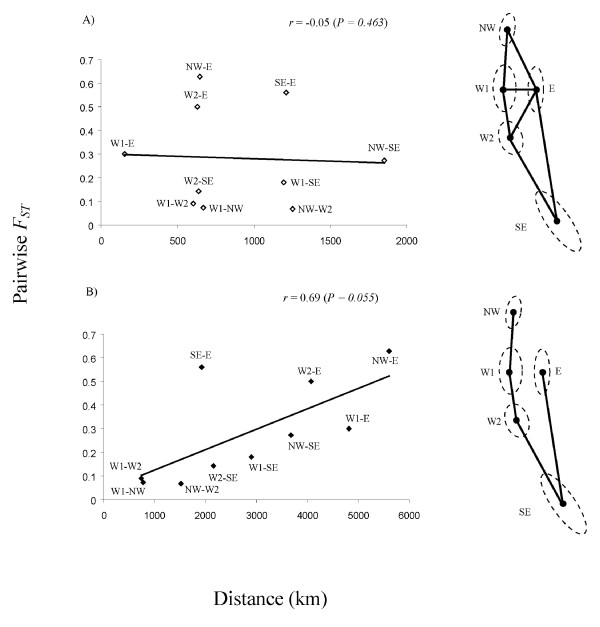
**Contrasts of pairwise genetic distance versus geographic distance between regional groups assuming marine and trans-andean dispersal**.

Mantel tests supported the marine realm as the most important route of dispersal into eastern Patagonia (Figure 7). A comparison of pairwise *F*_*ST *_versus geographic distance yielded a strong positive relationship when distance was modeled according to a scenario of marine dispersal (*r *= 0.69, *P = 0.055*). By contrast, direct distance between regions was poorly correlated with genetic distance (*r *= -0.05, *P *= 0.463).

### Drainage reversals

We conducted several hierarchical AMOVAs using control region sequences. First, when all collection sites (lake or river) were considered independently, 61% of the total variance in sequences was explained by differences among collection sites and 39% by variation among individuals within sites (Table 2). Grouping the 36 lakes/river collections into 17 basins explained nearly 30% of the total variance (Table 2) suggesting that populations from different lakes within river systems are more similar to each other than they are to populations in other river drainage systems. Thirdly, pooling samples according to their historical (pre-LGM) oceanic drainage (Pacific vs. Atlantic) explained approximately four times more variance than pooling them into present-day drainage (15.6% vs. 3.7%, Table 2) suggesting some of the systems that currently drain into the Pacific Ocean may historically have drained into the Atlantic Ocean.

### The Coastal Cordillera

We conducted an AMOVA considering only coastal and Andean river basins within latitudes 34°S and 40°S in Chile. This analysis revealed that grouping samples according to collection site (coastal vs. interior, with coastal being west and interior being east of the Coastal Cordillera) explained approximately twice as much genetic variation (13.93%) than grouping samples according to where the headwaters lie (Andean vs. coastal) (6.64%). This finding suggests that downstream populations of Andean systems in this region of Chile are more similar to populations of other coastal rivers than to upstream populations within their own river systems and suggests that the Coastal Cordillera may represent a partial barrier to geneflow (Table 2).

There were marked differences between coastal and Andean populations in Chile, particularly at mid-latitudes. Western Andean populations (Valdivia basin) are much more closely allied with populations east of the Andes at the same latitude (Limay & Manso river basins) than with a western coastal collection site within the same basin (Rio Valdivia) (Fig 6).

### Demography

**Figure 8:**
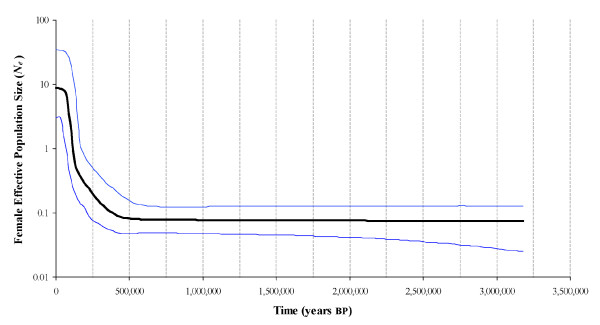
**Historical estimates of female effective population size through time constructed using the Bayesian skyline model **[49,50]**based on mitochondrial control region haplotypes**.

Demographic reconstructions based on control region haplotypes revealed long-established maternal effective population sizes which coalesce at approximately 3.2 million years BP (Figure 8). Effective population size appears to have remained relatively constant until roughly 0.5 million years BP when population size rapidly increased several orders of magnitude [100× (60×-190×)] to reach contemporary levels.

## Discussion

Of the potential vicariant processes associated with historical shifts in landscape and climate of ancient Patagonia, only mountain orogeny appears to have had significant influence on the distribution and genetic structure of *G. maculatus*. Effective population size increased through the past 0.5 Ma despite repeated periods of glacial advance and retreat. The role of Quaternary climatic cycles appears restricted to northern drainages where river drainage diversion swapped representatives of two divergent lineages. Otherwise, genetic structure appears to be a consequence of the variations in the life history of *G. maculatus*, responding to isolation from the sea (landlocking) or to other factors limiting female dispersal.

### Evolutionary Structure

Contemporary *G. maculatus *populations form several distinct matrilineal lineages whose phylogeographic signal suggests a probable pattern of dispersal and diversification within Patagonia. Extant populations are most likely derived from coastal populations in northern Chilean Patagonia. This region contains most of the current genetic diversity. One haplogroup (4) is common on the Atlantic and Pacific coasts, as well as in the Beagle Channel. Two sub-groups of haplogroup 4, sub-groups b and c, are almost entirely restricted to Andean locations, and the latter is more common in the north and found in both western and eastern drainages.

These patterns, combined with evidence of Isolation by Distance (IBD) under a scenario of marine dispersal (Mantel tests), suggest that *G. maculatus *began to diversify in systems of Northern Chilean Patagonia and subsequently spread south along the Pacific coast, colonizing adjacent coastal systems via stepping-stone dispersal, eventually reaching Atlantic systems via the southern tip of South America. On the Atlantic side, range expansion and diversification likely continued northward and westward. Haplogroup 4 was likely responsible for establishing the widespread distribution of *G. maculatus *on the eastern side of the Andes, given its current distribution and abundance. The pattern of expansion thus contrasts with those of other co-distributed, but freshwater-limited species such as *Percichthys trucha *and the closely related *G. platei *whose widespread contemporary distributions more strongly reflect the effects of vicariant separation initiated by mountain uplift [6,7]. Within a more recent timeframe, the invasive, diadromous Chinook salmon has followed a route to Argentina similar to that of *G. maculatus *after escaping from aquaculture pens in Pacific Chilean waters [52].

The strong matrilineal structure found in control region sequences suggests limited female dispersal among populations, but does not indicate whether male dispersal has also been restricted or if haplogroups represent only female philopatry. Contrasting genetic signatures between nuclear and mitochondrial markers are commonly employed to evaluate alternate explanations, and disagreement between marker classes is taken as evidence for sex-biased dispersal. However, in the present case, supplemental analysis at a single variable nuclear locus failed to provide a clear answer. The resulting nuclear gene tree was paraphyletic with respect to each haplogroup (Figure 4), a result which could be explained by inter-lineage hybridization via male-mediated gene flow or by the persistence of ancestral polymorphisms in isolated populations because of incomplete lineage sorting. Male-biased dispersal has been recognized in several species of fishes [53-55], and considering that polygamous mating strategies are thought to encourage male-biased dispersal [56], it seems plausible to consider that such a pattern could arise in a broadcast spawner such as *G. maculatus*. The large differences between coastal and Andean populations in Chile, however, are likely due to restricted dispersal by both sexes, as many of the Andean rivers and lakes are landlocked or are far from the sea with populations that are non-migratory [38,39]. Physical isolation or landlocking has been recognized several times as an important mechanism of diversification within galaxiids at both population and species levels [57-59]. Further investigation will be required to fully understand the biological explanations of genetic structure revealed by mitochondrial signal. In particular, several additional, independent nuclear loci are required in order to provide the statistical framework to confidently distinguish between instances of secondary contact and incomplete lineage sorting [60]. Our laboratory is currently developing suitable primers to address this problem.

South American populations of *G. maculatus *appear to show stronger regional genetic structuring than do populations in the eastern Pacific. In addition to our findings, Waters et al. [29] reported unexpectedly high estimates of genetic divergences at *cytochrome b *between the Malvinas (Falkland) islands and mainland populations. Zattara & Premoli [61] also found genetic signatures consistent with restricted geneflow between adjacent, landlocked populations in Argentina based on allozyme frequencies. By contrast, molecular studies in the eastern Pacific (i.e. New Zealand, Australia, Tasmania) suggest that *G. maculatus *is an uninhibited disperser capable of maintaining both regional and trans-oceanic gene flow. Baker and Lambert [62] found no evidence for genetic structuring between 4 populations from the Bay of Plenty, New Zealand using measures of geneflow based on several allozyme loci. Further phylogeographic analysis generalized this finding by reporting very little genetic structure between several broadly distributed locations throughout New Zealand [30]. DNA sequence data and allozyme-based studies also support recent trans-ocean dispersal between the eastern-Pacific continents [28,30]. These contrasting patterns suggest that the genetic structuring observed in Patagonian populations of *G. maculatus *may perhaps be unusual for the species.

### Drainage Reversals

The presence of the same haplogroups in eastern and western draining basins in the northern Patagonian Andean populations suggests that, in contrast to the coastal mountains in Chile, there has been substantial gene flow across the Andes. Two very divergent groups (1 and 4c) were found on both sides of the continental divide in this region. We suggest that rearrangement of the hydrological landscape during the Pleistocene, systems that experienced one or more drainage reversals between Pacific and Atlantic, is the mechanism most likely responsible for bidirectional, trans-Andean gene flow of *G. maculatus*, producing a distribution of genetic diversity that is better explained by ancient drainage direction than by current orientation. Along the latitudinal range of the Andes in Patagonia, there are several examples of river systems with headwaters originating in Argentina that bisect the mountains to drain into the Pacific. Continental ice during the LGM imposed a western barrier to aquatic systems east of the Andes forcing their flow into the Atlantic [20]. Subsequent periods of warming created large accumulations of melt water (paleolakes) at the eastern fringe of glacial mountain ice, dammed by terminal moraines in the east and the ice itself in the west. The collapse of the western barriers of these meltwater paleolakes as the ice melted resulted in a catastrophic drainage reversal from the Atlantic to the Pacific [18,20,63], with a significant flood of glacial melt water and any associated biota. For example, the Valdivia river system in Chile has its headwaters (Lago Lácar) in Argentina, and populations of *G. maculatus *in its Andean lakes are phylogenetically more closely allied with the northern populations of Argentina (Limay, Manso river basins) than with other nearby Chilean populations. The same mechanism is thought to be responsible for introducing eastern lineages of *G. platei *into the west [7]. In the present study, the presence of haplotypes of haplogroup 1 east of the Andes in Lake Quillén (Figure 5) suggests that dispersal also occurred west to east. The geological mechanism(s) that could have facilitated this exchange is not known. Perhaps individuals of *G. maculatus *occupied the headwaters of an ancient Pacific drainage which historically originated on the eastern side and were diverted along with the headwaters toward the Atlantic as glacial ice formed. Pleistocene-induced alterations in freshwater hydrology, especially river capture, have been very important influences on contemporary diversity within the Galaxiidae in New Zealand as well [64-69].

### Historical Demography

Long-term and widespread increases in effective population size despite repeated bouts of glacial advance throughout the Pleistocene suggest that contemporary populations of *G. maculatus *inhabiting post-glacial regions may have retreated to marine-based refugia during periods of global cooling. Bayesian skyline plots indicated only upward trends in female effective size over the past 0.5 Ma. Unvarying estimates of high haplotype, but low nucleotide diversity across sample locations are also consistent with genetic signatures of widespread population growth [70]. Access to stable marine environments would have released *G. maculatus *from the genetic purging via founder-flush cycles typically associated with freshwater refugia and would explain the contrasting patterns revealed in other co-distributed Patagonian freshwater fishes that showed evidence of at least one genetic bottleneck during the late Pleistocene [17]. A review of patterns of genetic diversity in the Palearctic and Nearctic revealed that this strategy appears to be widespread among diadromous fishes of the northern hemisphere [32,33,71], and is likely extendible to *G. maculatus *in the southern hemisphere. The reason for constant increase, however, is less clear. Perhaps the concomitant period of dramatic cooling was responsible for creating a scenario of ecological release in which severe conditions evicted most inhabitants from several Patagonian systems and allowed the uninhibited invasion of *G. maculatus *from several healthy marine-based sources. Interestingly, the period during which this invasion appears to have taken place immediately follows the coldest Patagonian glaciation approximately 0.7 Ma, which was responsible for dramatic declines in population sizes of the co-distributed freshwater galaxiid, *Galaxias platei*[17]. Perhaps similar glacial-induced extirpations affected other native species and provided the right motivation for *G. maculatus *to invade the eastern systems of Argentina.

## Conclusions

This study provides important insights into the potential mechanisms responsible for influencing the distribution and diversity of *G. maculatus *throughout Patagonia. Vicariant processes associated with mountain building and climate-induced drainage shifts appear to have been particularly influential in determining access routes of dispersal throughout Patagonia. Population sizes, however, remained very resilient in spite of repeated climatic shifts suggesting that marine environments played very important roles as glacial refugia on perhaps several occasions throughout the Quaternary. Several questions relating to the biology of *G. maculatus *also emerged based on the fine-scale phylogeographic structure, including the potential contributions of gender-specific dispersal and/or landlocking to population-level structuring. These broad-scale interpretations of genetic structure provide a much needed historical context within which studies of metapopulation dynamics can be properly interpreted, as well as *a priori *knowledge of regional diversity for phylogeographic and phylogenetic analyses.

## Authors' contributions

All authors contributed to the conception and design of the study, and all participated in sample collection. TSZ generated the molecular data, and TSZ and DER were responsible for data analysis. TSZ drafted the initial version of the manuscript, and DER and SJW were involved in subsequent versions of the manuscript. All authors reviewed it critically and have given final approval for publication.

## Supplementary Material

Additional file 1 **Maximum likelihood analysis of all mtDNA control region haplotypes from the present study and a subset of homologous haplotypes derived from New Zealand and Tasmanian populations of *Galaxias maculatus ***[30]. Phylogenetic analyses presented in Additional files 1 and 2 were conducted using partial isolates of control region sequences from [30] (AF240184-AF240339) to justify the use of a single sequence as an outgroup. File is Newick format. Click here for file

Additional file 2 **Maximum likelihood analysis of ~150 haplotypes derived from Tasmanian and New Zealand origins using partial isolates of control region sequences**. Phylogenetic analyses presented in Additional files 1 and 2 were conducted using partial isolates of control region sequences from [30] (AF240184-AF240339) to justify the use of a single sequence as an outgroup. File is Newick format. Click here for file

Additional file 3 **Geographic distances (km) between regional collection locations used in Mantel tests**. Geographic distances (km) between regional collection locations used in Mantel tests (see Table 1). Distances above diagonal are direct distances between regions and represent a trans-Andean dispersal model. Below the diagonal are distances measured assuming a marine-mediate route of dispersal (Figure 7). Click here for file

Additional file 4 **mtDNA haplotype frequency by collection location**. A detailed table listing the mtDNA haplotypes identified in the present study and the frequency that each occurred at each collection location. Click here for file
