# Supplementary material for: Surviving historical Patagonian landscapes and climate: molecular insights from Galaxias maculatus
Source: BMC Evol Biol. 2010 Mar 8;10:67. doi: 10.1186/1471-2148-10-67 (PMC2838892; doi:10.1186/1471-2148-10-67)
Supplement: Additional file 3 — Geographic distances (km) between regional collection locations used in Mantel tests. Geographic distances (km) between regional collection locations used in Mantel tests (see Table 1). Distances above diagonal are direct distances between regions and represent a trans-Andean dispersal model. Below the diagonal are distances measured assuming a marine-mediate route of dispersal (Figure 7). [file 1471-2148-10-67-S3.DOC]

**Supplemental material 3**. Geographic distances (km) between regional collection locations used in Mantel tests (see Table 1). Distances above diagonal are direct distances between regions and represent a trans-Andean dispersal model. Below the diagonal are distances measured assuming a marine-mediate route of dispersal (Figure 7).

|  | **NW** | **W1** | **E** | **W2** | **SE** |
| --- | --- | --- | --- | --- | --- |
| **NW** | - | 669 | 647 | 1253 | 1852 |
| **W1** | 782 | - | 155 | 602 | 1194 |
| **E** | 5600 | 4818 | - | 630 | 1212 |
| **W2** | 1523 | 741 | 4077 | - | 640 |
| **SE** | 3679 | 2897 | 1921 | 2156 | - |
