# Supplementary figures and images for: Surviving historical Patagonian landscapes and climate: molecular insights from Galaxias maculatus
Source: BMC Evol Biol. 2010 Mar 8;10:67. doi: 10.1186/1471-2148-10-67 (PMC2838892; doi:10.1186/1471-2148-10-67)

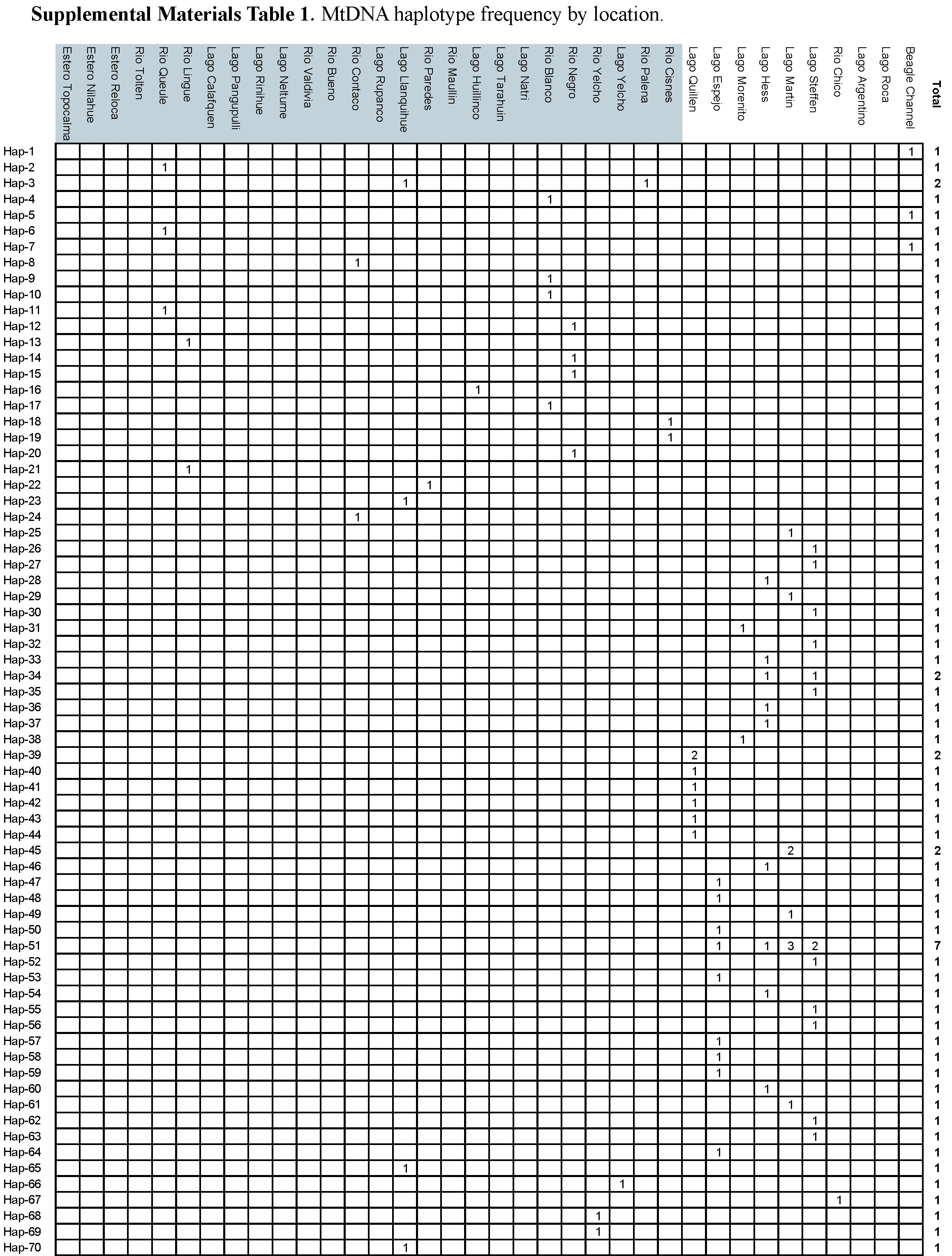


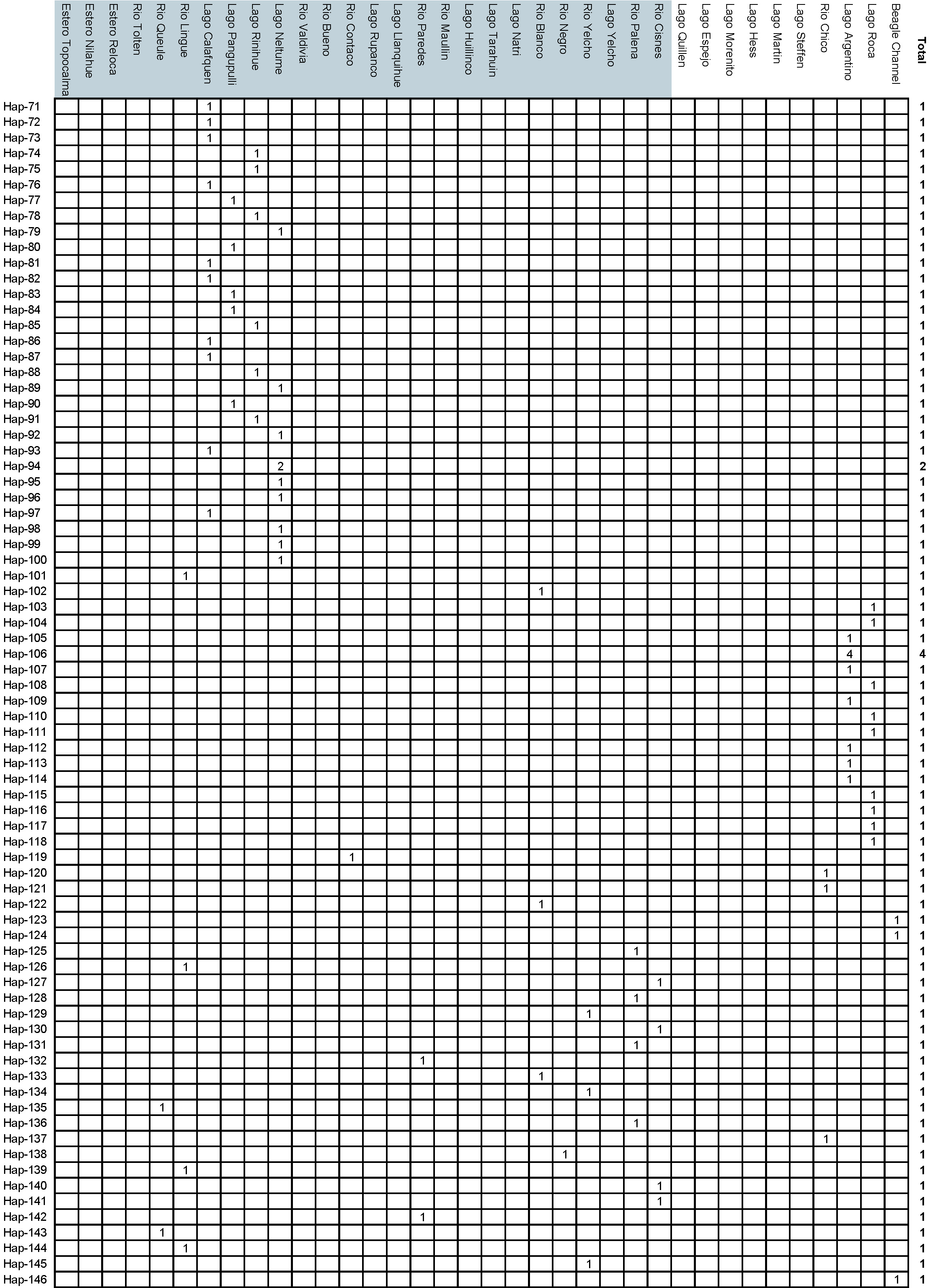


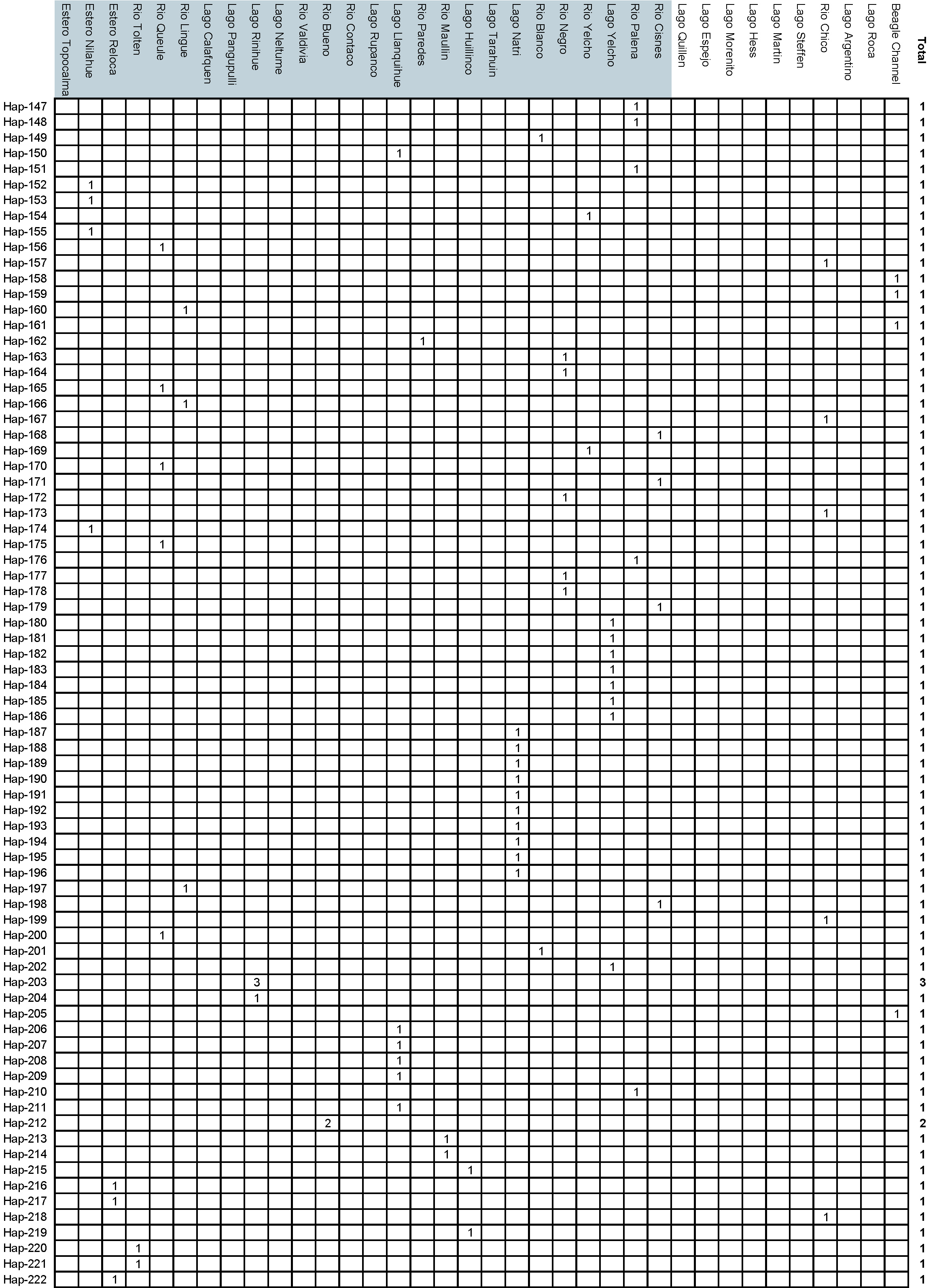


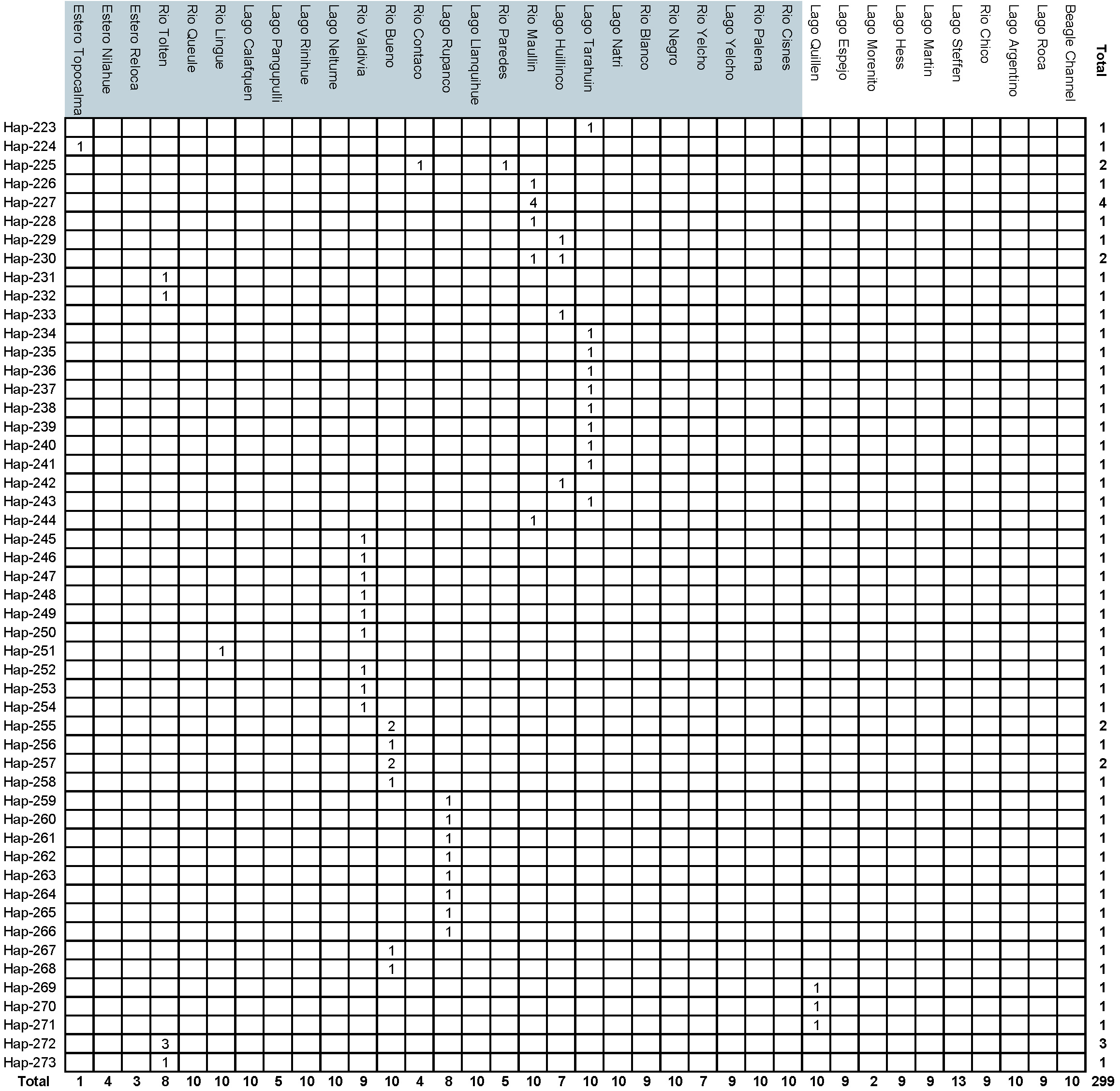

Supplement: Additional file 4 — mtDNA haplotype frequency by collection location. A detailed table listing the mtDNA haplotypes identified in the present study and the frequency that each occurred at each collection location. [file 1471-2148-10-67-S4.DOC]
